# Bovine respiratory syncytial virus and bovine coronavirus in Swedish organic and conventional dairy herds

**DOI:** 10.1186/s13028-014-0091-x

**Published:** 2015-01-13

**Authors:** Cecilia Wolff, Ulf Emanuelson, Anna Ohlson, Stefan Alenius, Nils Fall

**Affiliations:** Department of Clinical Sciences, Swedish University of Agricultural Sciences, PO Box 7054, SE-75007 Uppsala, Sweden; Växa Sverige, PO Box 210, SE-101 24 Stockholm, Sweden

**Keywords:** Bovine respiratory disease, Diarrhoea, Cattle, Disease prevalence, Disease incidence, ELISA

## Abstract

**Background:**

Infections with bovine respiratory syncytial virus (BRSV) and bovine coronavirus (BoCV) are endemic to the cattle populations in most countries, causing respiratory and/or enteric disease. It has been demonstrated that herds can remain free from these infections for several years also in high prevalence areas. Organically managed (OM) dairy herds have been shown to have lower seroprevalence of both viruses compared to conventionally managed (CM) herds. The objective of this study was to challenge the hypothesis of a lower occurrence of BRSV and BoCV in OM compared to CM dairy herds.

In November 2011, May 2012 and May 2013 milk samples from four homebred primiparous cows were collected in 75 to 65 OM and 69 to 62 CM herds. The antibody status regarding BRSV and BoCV was analysed with commercial indirect ELISAs. Herds were classified as positive if at least one individual sample was positive.

**Results:**

The prevalence of positive herds ranged from 73.4% to 82.3% for BRSV and from 76.8% to 85.3% for BoCV among OM and CM herds, over the three sampling occasions. There was no statistically significant difference between OM and CM herds at any sampling occasion. The incidence risk of newly infected herds did not differ statistically between OM and CM herds at any sampling occasion, neither for BRSV nor for BoCV. The incidence of herds turning sero-negative between samplings corresponded to the incidence of newly infected. Bulk tank milk (BTM) samples were also sampled in the herds and analysed. Several herds were negative on individual samples but positive in BTM. Herd-level data on production, health and reproduction were retrieved from VÄXA Sweden and the study herds were representative of the source population.

**Conclusion:**

There was no difference in prevalence of or incidence risk for BRSV or BoCV between Swedish OM and CM herds. Because the incidence of herds becoming seropositive was balanced by herds becoming seronegative it should be possible to lower the prevalence of these two infections among Swedish dairy cattle herds if biosecurity is improved.

**Electronic supplementary material:**

The online version of this article (doi:10.1186/s13028-014-0091-x) contains supplementary material, which is available to authorized users.

## Background

Infections with bovine respiratory syncytial virus (BRSV) [1-4] and bovine coronavirus (BoCV) [2,5] are endemic in the cattle populations in most countries. It has been demonstrated that a cattle herd can remain free from these infections for several years [6], even when located in high prevalence areas [7] and in close proximity to herds experiencing an (BRSV) outbreak [8]. Herds may become antibody negative to any of these infections within a few years provided the virus is not re-introduced into the herd [6,8].

BRSV commonly cause respiratory disease, particularly in calves. Disease can be caused by BRSV only or in combination with other viruses (e.g. BoCV) or secondary bacterial infection [9-11]. BoCV also causes enteric disease, in particular calf diarrhoea [12], and is the causative agent of Winter Dysentery, outbreak of diarrhoea, in adults [13]. There have been reports of BoCV as the single agent in outbreaks of respiratory disease as well [14,15]. In addition to impaired animal welfare due to illness, these infections may cause losses to production by reduced weight gain [16,17], reduced milk yield [4,18], increased bulk tank [19] and individual [18] milk somatic cell counts.

After infection with BRSV or BoCV animals will remain seropositive for several years. This was demonstrated by Bidokti *et al.* [20] who found herd where the older cows were sero-positive while the younger cows were sero-negative, i.e. there had been no virus circulating for several years. Maternal antibodies remain detectable for approximately 6 months [13,21], i.e. a never-infected heifer will be seronegative at the time of first calving. Both milk and blood samples can be used to assess the serological status of cattle [22]. When the herd‘s status is based on a bulk tank milk (BTM) sample, which is convenient e.g. for screening a population, the result will reflect the long term, i.e. up to the life-span of the oldest cows, history of the herd. However, if primiparous homebred cows are sampled, the results will give a more accurate description of the recent, i.e. the life-span of the tested cows, history of the herd.

Although these viruses may spread during the warmer seasons, seroconversion with or without an outbreak of clinical disease is more frequent during the housing season (autumn and winter) [6,23,24]. There is however, still a knowledge gap concerning what the most important routes for virus transmission between herds are. A few studies from the Nordic countries have studied risk factors for herds to be and to become seropositive to BRSV and BoCV. Risk factors at herd level have included a short distance to nearest herd, not providing boots to visitors, large herd size and a high density of cattle in the area [5,7,25,26].

A recent study found that organically managed (OM) dairy herds had significantly lower seroprevalence of both BoCV and BRSV compared to conventionally managed (CM) herds. However, the study could not explain the reason for the differences in the two production systems [20]. Although the difference was statistically significant, the study was made with a relatively small sample of herds. It would be beneficial for the organic as well as the conventional dairy production if these results were validated and studied further. Moreover, the Swedish dairy industry has over the last decade undergone rapid structural changes with increasing herd sizes and decreasing herd numbers. Simultaneously, the proportion of dairy cows under OM management has increased from 6% in 2005 to 13% in 2011 [27,28]. This also calls for a new assessment of disease occurrence in the two management systems. The objective of this study was therefore to challenge the hypothesis of a lower occurrence of BRSV and BoCV in OM compared to CM dairy herds and, specifically, to compare the herd prevalence and incidence.

## Methods

This was a prospective longitudinal observational study including OM and CM dairy herds. The unit of interest was herd.

### Study population

The sampling frame was all dairy herds with a yearly average herd size of at least 50 cows and enrolled in the Swedish Official Milk Recording Scheme. The inclusion criterion of a yearly average herd-size of at least 50 cows was chosen to exclude small herds which do not represent the “future” herd. Geographically, all counties except for most southern Skåne were included. Skåne was excluded because it was known that there are very few BRSV and BoCV negative herds in this region. OM herds were defined as herds with a dairy production certified according to the standards by the association KRAV (www.krav.se), the Swedish member of the International Federation of Organic Agriculture Movements.

The desired size of the study group was 100 OM and 100 CM herds which was as many as the project would be able to manage. The number of invited herds was based on previous experiences of about 30% willingness among Swedish dairy farmers to participate in similar observational studies. A simple random sample of 400 CM herds from the 1800 in the sampling frame, and all eligible OM herds (n = 244) were sent a written invitation to the study in May 2011. In total, 75 (31%) and 69 (17%) of farmers with OM and CM herds, respectively, agreed to participate in the project. Participants gave a written permission to access the herd’s data from the milk recording scheme.

### Data collection and management

#### Milk samples and analysis

Study herds were sent instructions, material and protocol for sampling in November 2011, May 2012, and May 2013. These occasions were chosen to include as many stall periods as possible during the project. At each sampling occasion, milk from four homebred primiparous cows and BTM were sampled into test tubes with 1.5 mg of the preservative agent Bronopol. Samples were returned to the National Veterinary Institute by prepaid mail where they were stored at −20°C until analysed.

The antibody status regarding BRSV and BoCV was analysed with the commercial indirect ELISAs (Svanovir BRSV-Ab and Svanovir BCV-Ab, Boeringer Ingelheim Svanova, Uppsala, Sweden). The optical density (OD) of samples was corrected by subtraction of negative control OD, and the percent positivity (PP) value was calculated as the corrected OD divided by the corrected OD for positive controls and multiplied by 100. The samples from individuals were classified as negative if PP < 10, following the manufacturer’s guidelines, and bulk tank samples if PP < 5. The sensitivity and specificity of the ELISA test kit was 94% and 100%, respectively, for BRSV and 84.6% and 100%, respectively, for BoCV, according to the manufacturer.

After each completed sampling occasion, the farmers were sent information on their herd’s serological status and information about basic biosecurity measures. A lottery ticket (value approx. 3 euro) was enclosed to the letter.

### Statistical analyses

#### Samples from individuals

Antibody status as measured in individual milk samples was used as an indicator of the herd’s recent infection status regarding the two viruses. Herds were defined as antibody positive if at least one of four samples from individual cows was positive. A stricter definition of positive herd where all four individual samples had to be positive was also applied. To assess any difference in antibody status between OM and CM herds, the prevalences of positive herds at each sampling occasion among OM and CM herds, respectively, were calculated with exact binomial confidence intervals using the package “binom” for R [29]. Data management and analysis was performed in R version 3.0.1 [30].

Further, the incidence risk with exact binomial confidence intervals was calculated for the samplings in 2012 and 2013, for OM and CM herds, respectively. The period at risk was from the last sampling occasion, i.e. 6 months and 12 months, respectively.

#### Bulk tank milk samples

To explore if there were differences in antibody level in the bulk tank milk between the CM and OM herds, the results from the BTM samples (PP values) were categorised into six groups; PP < 5, 5 ≤ PP < 10, 10 ≤ PP < 30, 30 ≤ PP < 60, 60 ≤ PP < 100, and 100 ≤ PP. The frequencies of OM and CM herds in each group were tabulated for each year and virus.

#### Herd data

Data on herd production, health and reproduction were retrieved from the Swedish Official Milk Recording Scheme database managed by VÄXA Sweden for all selected herds, i.e. including also the invited but non-participating herds. The information included averages of routinely collected parameters for the 12 months before the start of the study.

Herd parameters were described and tested for difference between the OM and CM herds that participated in 2011 i.e. entered the study, using a two-sided Wilcoxon rank-sum test or Fischer exact test. To study how the study group characteristics were affected from drop-out herds leaving the study, herd parameters were described for the subsample of OM and CM study herds that completed the last sampling in 2013. To examine the representativeness of the study herds, herd parameters were also described for all invited OM and CM that did not participate in the study.

## Results

The number of study herds per year was 144, 132, and 127 in 2011, 2012 and 2013, respectively (Table 1). No new herds entered the study after 2011, however one herd was sampled in 2011 and 2013 but not in 2012. All herds had individual samples but in 2012 and 2013 the number of bulk tank milk samples was 131 and 126, respectively.

**Table 1:** **Antibody status in a selection of Swedish organically and conventionally managed dairy herds determined from milk samples from four primiparous cows at three sampling occasions**

	**Autumn 2011**		**Spring 2012**		**Spring 2013**		**Total all samplings**	
	**Organic (n = 75)**	**Conv. (n = 69)**	**Organic (n = 68)**	**Conv. (n = 64)**	**Organic (n = 65)**	**Conv. (n = 62)**	**Organic**	**Conv.**
BRSV								
Negative herds (n)	14	18	14	13	14	11	42	42
Prevalence (%) of positive herds (95% CI)^a^	81.3 (70.7, 89.5)	73.4 (61.9, 83.7)	79.4 (67.9, 88.3)	79.7 (67.8, 88.7)	78.5 (66.5, 87.7)	82.3 (70.5, 90.8)		
Herds going from negative to positive between sampling occasions (n)	-	-	3	7	3	4	6	11
Incidence risk (%) of herd becoming antibody positive (95% CI)^a^	-	-	21.4 (4.7, 50.8)	38.9 (17.3, 64.3)	23.1 (5.0, 53.8)	36.4 (10.9, 69.2)		
Herds going from positive to negative between sampling occasions (n)	-	-	3	2	4	4	7	6
Prevalence (%) of herds with four of four positive individual samples (95% CI)^a^	62.7 (50.8, 73.6)	50.7 (38.4, 63.0)	66.2 (53.7, 77.2)	68.7 (55.9, 79.8)	66.2 (53.4, 77.4)	71.0 (58.1, 81.8)		
BCoV								
Negative herds (n)	11	16	11	14	14	10	36	40
Prevalence (%) of positive herds (95% CI)^a^	85.3 (75.3, 92.4)	76.8 (65.1, 86.1)	83.8 (72.9, 91.6)	78.1 (66.0, 87.5)	78.5 (66.5, 87.7)	83.9 (72.3, 92.0)		
Herds going from negative to positive between sampling occasions (n)	-	-	3	4	2	6	5	10
Incidence risk (%) of herd becoming antibody positive (95% CI)^a^	-	-	27.3 (6.0, 61.0)	26.7 (7.8, 55.1)	20.0 (2.5, 55.6)	46.2 (19.2, 74.9)		
Herds going from positive to negative between sampling occasions (n)	-	-	3	2	6	3	9	5
Prevalence (%) of herds with four of four positive individual sample (95% CI)^a^	78.7 (67.7, 87.3)	68.2 (55.8, 78.8)	69.1 (56.7, 79.8)	64.1 (51.1, 75.7)	67.7 (54.9, 78.8)	66.2 (53.0, 77.7)		
Herds negative to both BRSV and BCoV (n)	3	9	5	6	7	4	15	19

A herd was defined as positive at the sampling occasion if at least one cow was antibody-positive to Bovine Respiratory Syncytial Virus (BRSV) or Bovine Corona Virus (BCoV), respectively. Analysis was conducted by using indirect ELISA.

^a^Exact binomial confidence intervals.

### Samples from individuals

Based on the test results from sampled individuals the prevalence of BRSV positive herds ranged from 73.4% to 82.3% over three sampling occasions among OM and CM herds (Table 1). In 2011, the prevalence of positive herds was lower in CM herds, and in 2012 and 2013 in OM herds. For BoCV the prevalence estimates ranged from 76.8% to 85.3% positive herds over three sampling occasions among OM and CM herds (Table 1). In 2011 and 2012 the prevalence of positive herds was lower in CM herds and in 2013 in OM herds. The confidence intervals of OM and CM herds’ prevalences were overlapping all years and it was therefore concluded that there was no statistical difference in the prevalence of BRSV or BoCV between OM and CM herds.

The prevalence of BRSV-positive herds decreased to 50.7-71.0% and for BoCV to 64.1-78.7% with the stricter the criteria of four out of four positive individual samples for a herd to be classified as positive at the sampling occasion (Table 1).

The incidence risk of newly infected herds, i.e. herds that went from negative to having at least one of four primiparous cows with a positive test result, did not differ statistically between OM and CM herds in any study year, neither for BRSV nor for BoCV (Table 1). There were in total 12 herds that were antibody-negative to both viruses in 2011 (8.3%), and 11 herds in both 2012 (8.3%) and 2013 (8.6%) (Table 1).

### Bulk tank milk samples

Not more than 7, 3 and 4 herds were BRSV negative and 5, 6, and 3 herds were BoCV negative in 2011, 2012 and 2013, respectively (Table 2). All these herds negative in BTM were also negative based on the individual samples. Further, for both viruses, in a high proportion of all herds with BTM PP < 30, all individual samples were negative. And, on the contrary, in a high proportion of herds with BTM PP ≥ 100, all individual samples were positive (Table 2).

**Table 2:** **Distribution of herd bulk tank milk antibody percent positivity (PP) values in organically (OM) and conventionally managed (CM) Swedish dairy herds**

	**2011**		**2012**		**2013**		**Negative herds** ^**a**^ **(%)**
**BRSV**	**OM (n = 75)**	**CM (n = 69)**	**OM (n = 68)**	**CM (n = 63)**	**OM (n = 65)**	**CM (n = 61)**	
PP < 5	2 (2)	5 (5)	1 (1)	2 (2)	3 (3)	1 (1)	100
5 ≤ PP < 10	1 (1)	0 (0)	2 (2)	3 (3)	3 (3)	3 (2)	92
10 ≤ PP < 30	4 (3)	4 (4)	5 (4)	3 (2)	4 (3)	3 (2)	78
30 ≤ PP < 60	5 (2)	5 (3)	5 (3)	3 (3)	4 (2)	4 (3)	62
60 ≤ PP < 100	18 (2)	20 (5)	41 (3)	34 (3)	22 (2)	28 (3)	11
100 ≤ PP	45 (4)	35 (1)	14 (1)	18 (0)	29 (1)	22 (0)	4
BCoV							
PP < 5	3 (3)	2 (2)	3 (3)	3 (3)	1 (1)	2 (2)	100
5 ≤ PP < 10	0 (0)	1 (1)	1 (1)	0 (0)	2 (2)	0 (0)	100
10 ≤ PP < 30	4 (4)	2 (2)	1 (1)	2 (1)	3 (3)	4 (3)	88
30 ≤ PP < 60	1 (0)	5 (3)	11 (3)	10 (4)	3 (3)	2 (1)	44
60 ≤ PP < 100	27 (3)	17 (5)	32 (2)	34 (5)	18 (2)	21 (4)	14
100 ≤ PP	40 (1)	42 (3)	20 (1)	14 (1)	38 (3)	32 (0)	5

The figures in brackets indicate the number of herds in each group where samples from all four individual were negative (PP < 10).

^a^Total proportions, i.e. over three sampling occasions, of sampled herds where also the four individuals were negative (PP < 10).

A higher number of herds were defined antibody positive based on analysis of BTM samples compared to individual samples, and the majority of the BTM positive herds had antibody levels of at least PP 60, i.e. clearly above the cut-off value. There were 26, 24, 22 herds negative to BRSV based on individual samples but positive based on BTM the sample in 2011, 2012, and 2013, respectively. For BoCV there were 23, 20 and 21 herds that were negative based on individual samples but positive based on the BTM sample at the same sampling occasions.

### Herd data

At study start the OM study herds were larger, had higher BTM somatic cell counts, lower incidence of veterinary treated disease events but also a lower production compared to the CM study herds (See Additional file 1: Table S1).

Herd data for herds entering the study and herds completing the study were judged as comparable (See Additional file 1: Table S1). The characteristics of the herds entering the study were judged as comparable to the invited herds not entering the study (See Additional file 1: Table S1).

## Discussion

In this study, there was no difference in incidence or prevalence of BRSV or BoCV antibody positivity between OM and CM herds. This disagrees with previous results from Sweden where the mean seroprevalence of antibodies in individual cows for BRSV and BoCV was found to be lower in OM dairy herds compared to CM [20]. Ohlson *et al.* [25] could not report any differences between OM and CM herds with respect to BRSV and BoCV seropositivity, which agrees with the results in the present study. However, that study included only a small sample of OM herds.

The number of Swedish OM dairy cows increased from 22,321 in 2005, when the herds in Bidohkti *et al.* [20] were recruited, compared to 44,133 in 2011 while during the same time period, the total number of dairy cows decreased from 393,263 to 346,495 [27,28]. A possible explanation to the different results in the current study compared to [20] could be that the characteristics of the farmers with OM and their herds as a group have changed from 2005 to 2011. Such a change of the “typical organic farmer” over time and in relation to market demand and political decisions, e.g. how incentives for organic farming might have developed from ideological for early converters to more financial for later, was discussed for Denmark [31]. Interestingly, the total number of OM herds going from positive to negative was higher compared to CM herds, but the opposite was true for herds going from negative to positive status (Table 1). This could be hypothesised to be a result of better implementation of the biosecurity advice to participants by the farmers with OM.

Although OM herds were overrepresented in the study group, the overall prevalence estimates, as well as for OM and CM separately, are in concordance with previous studies where herd prevalence of BRSV in BTM samples was 41-51% in northern Sweden and 84-89% in the most southern parts [3], and 85% in a study with seven southern and northern counties [25]. For BoCV herd prevalence was reported as 70-74% to 95-100% in BTM samples depending on region [5] and 81% in a study with southern and northern counties [25]. The national prevalence of these two infections seems not to have changed a lot from the late 1990-ies. It would be interesting to further explore how changes of routines or characteristics of dairy herds during a bit more than a decade might be associated with the prevalence of the two infections. For example, if the greatly improved biosecurity in many farms might have been offset by poor routines in others during the last decade. The current sampling frame included all counties of Sweden but the Skåne county which from previous research was known to have a herd prevalence near 100% and therefore considered as of less interest in this study. In a screening of bulk tank milk samples in 2013, including 95% of all Swedish dairy herds, the herd prevalence in Skåne was 99% (personal communication A Ohlson).

Every year several herds became antibody negative, and in other words gained BRSV and/or BoCV free status, but the incidence of number of herds becoming positive equalled out the number of herds clearing the infection. There were also a considerable number of herds with negative primiparous cows but positive BTM, i.e. herds in which there had been no virus circulating for at least 2–3 years. Apparently, there are good chances for a herd to free from these infections if good biosecurity is practiced and the virus is not re-introduced. This result agree with a Norwegian survey where the incidence of herds becoming BRSV positive (at least one antibody positive animal) and negative after six months was 33% and 42%, respectively [8]. A high turnover of positive herds was also found in a Swedish study over several years [6]. If introduction of BRSV and BoCV to a herd can be eliminated or kept at lowest possible level, it would be possible to significantly reduce the prevalence of these infections in the Swedish dairy population.

Many of the herds with positive BTM samples, but with a low PP value, i.e. <30 had only negative individuals. This indicates that there had been no virus circulating in the herd the last 2 to 3 years (Table 2) but was rather due to antibodies from older cows who experienced the infections several years ago. This finding agrees with Ohlson *et al.* [6] where all sampled primiparous cows in herds with a BTM sample PP value <30 were negative. We would therefore recommend sampling milk from young homebred cows over BTM samples in prevalence studies/screenings, because of the advantage that herds where there has been no recent active infection can be identified. To save analysis expenditure individuals’ samples, in addition, can be pooled for analysis without significant loss of sensitivity or specificity at herd level [22]. Also, the prevalence of positive herds decreased when “positive” was defined as four of four positive individuals (10 ≤ PP) but the difference in prevalence using this stricter definition was not significant (Table 1). This supports that BRSV and BoCV virus have efficiently spread in the herd after introduction and results in a subsequent high within-herd seroprevalence. With a herd size of 70, the test characteristics of the two ELISAs used, and a cut-off point for positive herd of one positive out of four tested individuals, the HSe for BRSV is 93% and 99% at a within-herd prevalence of 50% and 70% respectively. For BoCV the HSe is 90% and 98% at a within-herd prevalence of 50% and 70%, respectively. HSp is 100% for both tests as calculated with the web based tool [32] using the hypergeometric distribution. The herd sensitivity of the serological test can be increased by increasing the number of sampled individuals per herd; however, with a high within herd prevalence this becomes less influential.

A limitation of the study is the less than perfect sensitivity of the two ELISA tests used. Because the aim was to compare OM and CM herds, the true prevalence or incidence risk was not estimated. Any misclassification bias due to test characteristics should be the same in the two groups. Further, the different lengths of period at risk for the incidence estimates at the second and third sampling occasions was not optimal, because it made comparisons between sampling occasions impossible. This, however, does not influence the comparison of OM and CM herds at the same sampling occasion. However, this comparison could have been improved with a larger number of study herds. For example, the number of herds going from positive to negative or the opposite was now low and the confidence intervals of estimates wide. It would also have been beneficial with a longer total study period, thus including more than two housing periods, and more frequent sampling to explore how herds’ status change over time with better precision.

We consider the OM and CM study herds as representative of the OM and CM dairy herds in Sweden, because their production and health parameters did not differ substantially from the invited but non-participating herds (Additional file 1: Table S1). However, the study herds could still be biased towards more engaged and skilful farmers compared to non-participants and a higher response rate would have been desirable to minimize this risk. This is particularly valid for the CM herds, where the response rate was 17% compared to 31% for the OM herds. This could mean that the farmers with OM more accurately represent the OM population, while a possibly stronger bias towards a stratum of “interested, progressive and better” farmers with CM could be envisioned. The invited CM herds were a random sample meaning the herds entering the study represented 4% of the eligible herds, while the invited OM herds were a census, thus the participating herds represented 31% of the eligible herds. Because the study included three sampling occasions the possible increase in response rate after reminders, e.g. by telephoning non-responders, was weighted against the risk of losing these additional participants to the next sampling occasion. The inclusion criterion of a yearly average herd-size of at least 50 cows meant that 1,960 of the 4,022 dairy herds enrolled in milk recording in 2011 were not eligible for the study and the results should only be extrapolated to the larger herds. We judged that the study herds that participated to the last sampling occasion were comparable to study herds that participated at the start, i.e. herds leaving the study did not change the characteristics of the study group (Additional file 1: Table S1).

## Conclusions

There was no difference in prevalence of or incidence risk for BRSV or BoCV between organically and conventionally managed dairy herds in Sweden. The incidence risk of herds becoming seropositive was balanced by herds becoming seronegative, but with improved biosecurity it should be possible to lower the prevalence of these two infections among Swedish dairy cattle herds.

## Additional file

Additional file 1: Table S1. Herd characteristics and production and health parameters at the time for the start of the study for organically managed (OM) and conventionally managed (CM) herds entering the study, the study herds completing the full study period and invited but non-participating herds.
