# Supplementary material for: Bovine respiratory syncytial virus and bovine coronavirus in Swedish organic and conventional dairy herds
Source: Acta Vet Scand. 2015 Jan 13;57(1):2. doi: 10.1186/s13028-014-0091-x (PMC4300160; doi:10.1186/s13028-014-0091-x)
Supplement: Additional file 1: Table S1. — Herd characteristics and production and health parameters at the time for the start of the study for organically managed (OM) and conventionally managed (CM) herds entering the study, the study herds completing the full study period and invited but non-participating herds. [file 13028_2014_91_MOESM1_ESM.docx]

**Additional file 1**

Herd characteristics and production and health parameters at the time for the start of the study for organically managed (OM) and conventionally managed (CM) herds entering the study, the study herds completing the full study period and invited but non-participating herds.

| Parameter (median (p10, p90) | OM herds entering study  (n = 75) | CM herds entering the study (n = 69) | OM herds participating at the end of the study (n = 65) | CM herds participating at the end of the study (n = 59) | Invited OM herds not entering the study (n = 163) | Invited CM herds not entering the study (n = 319) |
| --- | --- | --- | --- | --- | --- | --- |
| Average herds size (n cows) | 75.40 (57.14, 126.64) | 69.75 (53.15, 176.20) | 75.40 (54.14, 123.80) | 70.00 (53.19, 194.94) | 77.8 (54.2, 182.0) | 76.5 (52.4, 168.1) |
| Average milk production per cow (kg ECM) | 8887 (7291, 10136) ^a^ | 9675 (8221, 10691) ^a^ | 8916 (7616, 10199) | 9789 (8551, 10736) | 8536 (7347, 9854) | 9362 (7957, 10502) |
| BTMSCC^b^ (10^5^ cells per 100 ml) | 259.0 (168.2, 356.4) ^a^ | 220.0 (145.4, 322.8) ^a^ | 251.0 (168.2, 361.2) | 220.0 (144.4, 320.0) | 266.0 (186.0, 359.8) | 253.0 (166.0, 363.0) |
| Calving interval (months) | 12.89 (12.30, 14.16) | 13.15 (12.39, 14.11) | 12.89 (12.30, 14.12) | 13.13 (12.35, 14.16) | 13.03 (12.30, 14.34) | 13.28 (12.53, 14.63) |
| Average age 1^st^ calving (days) | 818.9 (767, 933) | 824.6 (766, 939) | 815.1 (767.0, 910.7) | 824.6 (763.9, 937.4) | 842.8 (787.1, 988.9) | 853.6 (777.5, 993.3) |
| Calf deaths day 1 to 60 (incidence %) | 2.080 (0, 6.14) | 1.440 (0, 6.52) | 2.06 (0, 6.92) | 1.42 (0, 7.17) | 2.30 (0, 6.03) | 2.22 (0, 7.27) |
| Calf deaths month 2 to 6 (incidence %) | 0.00 (0, 4.28) | 0.00 (0, 4.06) | 0.00 (0, 3.61) | 0.00 (0, 3.75) | 0.00 (0, 4.06) | 0.00 (0, 5.24) |
| Removed cows (cows per 100 cow-years) | 32.40 (23.93, 46.39) | 33.13 (23.88, 43.08) | 32.38 (23.18, 47.57) | 33.18 (23.67, 41.62) | 30.70 (19.70, 43.48) | 34.44 (23.86, 47.46) |
| Recorded disease incidence (%) | 18.72 (3.16, 43.06) ^a^ | 24.09 (8.69, 54.39) ^a^ | 18.72 (5.42, 42.20) | 24.09 (9.28, 52.06) | 18.17 (3.05, 44.38) | 19.86 (3.36, 44.79) |
| Main breed in the herd (n herds (%)) |  |  |  |  |  |  |
| Swedish Red | 22 (29) | 14 (21) | 21 (32) | 12 (20) | 34 (20) | 69 (22) |
| Swedish Holstein | 13 (17) | 16 (24) | 12 (20) | 16 (27) | 26 (16) | 77 (24) |
| Swedish Polled | 0 (0) | 0 (0) | 0 (0) | 0 (0) | 2 (1) | 0 (0) |
| Swedish Jersey | 1 (1) | 1 (2) | 1 (2) | 1 (2) | 0 (0) | 1 (0) |
| Mixed herd | 39 (52) | 35 (53) | 31 (48) | 31 (52) | 105 (63) | 170 (54) |

^a^ Significant difference (*P* < 0.05) at Wilcoxon rank-sum test between organic and conventional herds at the start of the study.

^b^ Bulk Tank Milk Somatic Cell Count.
